# Video-Based Human Activity Recognition Using Deep Learning Approaches

**DOI:** 10.3390/s23146384

**Published:** 2023-07-13

**Authors:** Guilherme Augusto Silva Surek, Laio Oriel Seman, Stefano Frizzo Stefenon, Viviana Cocco Mariani, Leandro dos Santos Coelho

**Affiliations:** 1 Industrial and Systems Engineering Graduate Program (PPGEPS), Pontifical Catholic University of Parana (PUCPR), Curitiba 80215-901, Brazil; 2 Graduate Program in Applied Computer Science, University of Vale do Itajai, Itajai 88302-901, Brazil; 3 Digital Industry Center, Fondazione Bruno Kessler, 38123 Trento, Italy; 4 Department of Mathematics, Computer Science and Physics, University of Udine, 33100 Udine, Italy; 5 Department of Electrical Engineering, Federal University of Parana (UFPR), Curitiba 81530-000, Brazil; 6 Mechanical Engineering Graduate Program (PPGEM), Pontifical Catholic University of Parana, Curitiba 80215-901, Brazil

**Keywords:** convolutional neural network, deep learning, self-DIstillation with NO labels (DINO), video human action recognition, vision transformer architecture

## Abstract

Due to its capacity to gather vast, high-level data about human activity from wearable or stationary sensors, human activity recognition substantially impacts people’s day-to-day lives. Multiple people and things may be seen acting in the video, dispersed throughout the frame in various places. Because of this, modeling the interactions between many entities in spatial dimensions is necessary for visual reasoning in the action recognition task. The main aim of this paper is to evaluate and map the current scenario of human actions in red, green, and blue videos, based on deep learning models. A residual network (ResNet) and a vision transformer architecture (ViT) with a semi-supervised learning approach are evaluated. The DINO (self-DIstillation with NO labels) is used to enhance the potential of the ResNet and ViT. The evaluated benchmark is the human motion database (HMDB51), which tries to better capture the richness and complexity of human actions. The obtained results for video classification with the proposed ViT are promising based on performance metrics and results from the recent literature. The results obtained using a bi-dimensional ViT with long short-term memory demonstrated great performance in human action recognition when applied to the HMDB51 dataset. The mentioned architecture presented 96.7 ± 0.35% and 41.0 ± 0.27% in terms of accuracy (mean ± standard deviation values) in the train and test phases of the HMDB51 dataset, respectively.

## 1. Introduction

Human action recognition (HAR) is an interdisciplinary field related to computer vision that seeks to analyze human motion, balance, postural control, and interactions with their environment. It comprises biomechanics, machine vision, image processing, data analytics, nonlinear modeling, artificial intelligence, and pattern recognition. It can be analyzed through bidimensional, deep, or thermal images or movement, sensors adapted to the body, or smartphones [1]. In this context, the movements and positions of body parts are used to recognize human actions in human model-based methods. However, to develop an applicable and efficient HAR system, researchers must analyze the diversity in human body sizes, postures, motions, appearances, clothing, camera motions, viewing angles, and illumination.

HAR has been studied due to its numerous applications in a wide range of domains and complexities, highlighting applications in safety, environmental monitoring, video surveillance [2,3], robotics [4], training and practical courses with immediate response [5], healthcare, specific medical diagnosis and fitness monitoring [6], biomechanical analysis approaches using data analytics [7], among others. The main challenges in HAR include (i) inference from nonexplicit poses and actions; (ii) different people can classify poses and actions differently; (ii) the possibility of partial occlusion of the body or objects involved in the scene; (iii) videos of questionable quality, such as blurring, and poor-quality sensors which generate noise in the data; (iv) large differences between the times of different actions; (v) no lighting or high brightness; and (vi) difficult acquisition of large-scale datasets [8].

With technological advances in smartphones, it has been possible to collect data from various types of sensors, including accelerometers, gyroscopes, microphones [9], and cameras [10], to measure activities of daily living but without the explicit interaction of users with acquisition devices, i.e., not interfering or disturbing the actions [1]. Using these datasets collected by sensors and developing artificial intelligence techniques can provide an advanced understanding of the image caption task for activity detection or recognition. However, these techniques are shown to be limited and dependent on the extractor, making their usefulness restricted to specific applications [11]. In this context, deep learning approaches begin to stand out due to their generalization capabilities and the fact that there is no need to model the extraction of manual characteristics [12].

The convolutional neural networks (CNN) [13] evaluated in this paper are a residual network (ResNet) depth 50 [14] and a bi-dimensional vision transformer (ViT) with long short-term memory network (LSTM) [15]. The performance indicator that helps us evaluate the classifiers is the accuracy measure. The main objective of this study is to evaluate a hybrid deep learning model of supervised and semi-supervised learning for HAR in red, green, and blue (RGB) videos applied to the human motion database (HMDB51). The focus is a deep learning architecture that proves to be feasible for application in a real-life scenario, in which the algorithm processing can follow the real rate of image capture. In summary, we make the following contributions:

(i) A systematic review of the literature was conducted on themes related to HAR;
(ii) A label smoothing technique was tested with a 3D ResNet-50 in the HMDB51;
(iii) A model based on a semi-supervised learning methodology was evaluated in the HMDB51;
(iv) The results analysis of the proposed deep learning approach are presented based on the accuracy indicator applied to the HMDB51.

The remainder of this paper is organized as follows: Section 2 introduces relevant works relating to HAR and the ideas that helped define this work. Section 3 presents the used database. Section 4 focuses on the methodology applied to ResNet-50, a CNN with a fully connected layer, and a 2D ViT with LSTM. Section 5 presents the experiments and results analysis. Section 6 concludes this paper and future directions of research.

## 2. Related Works

In this section, we provide a comprehensive introduction to previous studies in the related fields of HAR. To guide this process, a set of keywords was defined: “activity recognition”, “action recognition”, “behavior recognition”, “RGB (red, green and blue) video”, “single camera video”, “mono camera video”, “deep learning”, “neural network”, and “CNN”.

**Figure 1:**
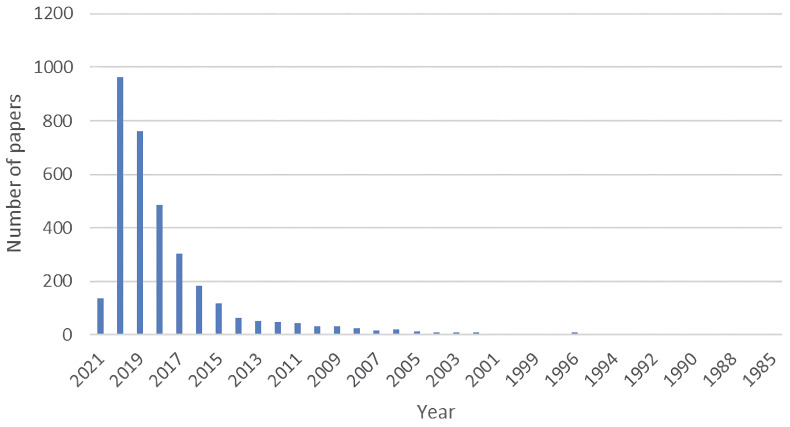
Annual distribution of consolidated papers from the three databases.

**Figure 2:**
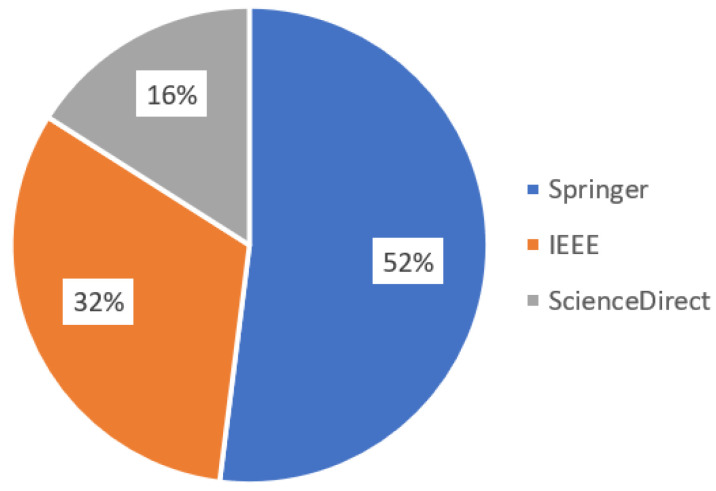
Percentage of papers selected by database from 2015 to 2021.

The research was carried out in a period ranging from 01/1985 to 01/2021 using three databases, IEEE (Institute of Electrical and Electronics Engineers) Digital Library, Science Direct, and Springer Link, obtaining a total of 4334 papers as illustrated in Figure 1. From the analysis of the data in Figure 1, studies published before 2015 were discarded due to the low distribution of articles in the early years and the constant evolution of technology, totaling 2952 documents, whose percentage distribution is shown in Figure 2.

Then, other exclusion criteria were applied in addition to the number of citations per article. These articles were classified into two groups. Both groups are related to RGB video image processing applied to HAR, group 1 is for articles using deep learning techniques, with 42 articles, and group 2 is for unsupervised learning, with 18 articles. Thus, 60 articles were included in the qualitative analysis.

In the recent literature, there are studies with several deep learning architectures [16], varying the type of pre-processing [17], input formats [18], artificial neural network configuration, memories, recurrences, filters [19], and final classification layers, among others [20]. However, there is still space to improve video classification when compared to image classification [21]. The 2D CNNs are still widely used [22] for the recognition of actions [22], and even though they cannot capture temporal characteristics, other complementary structures are proposed, such as optical flows [23], LSTM [24,25,26], and temporal groupings [27]. A complete review of human activity recognition using CNNs can be seen in [28].

Another frequently used strategy is that of streams, in which various types of input are processed in different networks, the most common is the two-stream network that processes RGB video frames in one and an optical stream in the other; Hao and Zhang employed this architecture [29]. The use of artificial intelligence models is growing in line with increased processing power, making deep learning applications increasingly popular [30]; these applications include time series prediction [31,32,33] and classification, especially in computer vision [34,35,36].

A structure that has been widely explored with the emergence of large datasets is 3D CNN, as described in Table 1. A disadvantage of this architecture is the high number of parameters, an order of magnitude greater than 2D CNNs, which often leads to overfitting [21]. Hara et al. [37] performed tests using 3D CNN applied to HMDB51, the University of Central Florida (UCF101) dataset, and the activity net dataset, but they did not obtain acceptable generalization results; however, while using Kinetics, those authors obtained a performance like that presented in the literature.

Recently, video databases for human activity recognition have started to get bigger, in the hundreds of thousands. Kinetics was proposed in 2019 [51] with 700 thousand labeled videos and Sports-1M in 2014 [52] with 1.1 million. Another alternative to a large labeled dataset is using a self-supervised or unsupervised learning method to extend the data universe without needing to go through the long labeling process [51].

ResNet and ViT are CNN-based models which are becoming popular given their high performance in classification tasks. Using ResNet-50, Wen, Li, and Gao [53] obtained accuracies of 98.95%, 99.99%, and 99.20% for fault diagnosis, outperforming other deep learning models. According to He, Liu, and Tao [54], the residual connections boost the performance of the neural nets. Xue and Abhayaratne [55] applied ResNet for the classification of COVID-19, and when they used 3D ResNet-101 an accuracy of 90% was achieved, which was better than other methods.

Li and He [56] proposed an improved ResNet, and by adjusting the shortcut connections, they obtained an accuracy of 78.63%, which was 2.85% higher than the original ResNet. These results were based on an evaluation using the CIFAR-10 dataset. On CIFAR-100, the accuracy of their method was 42.53%. The variation in the structure of the method was also studied by Paing and Pintavirooj [57], where a fast Fourier ResNet was proposed. Using a model based on ResNet-50, they achieve an F1-score of 0.95 for colorectal polyp adenoma dysplasia classification.

Using ViT, Wang et al. [58] evaluated the genitourinary syndrome of menopause. Considering optical coherence tomography images, they obtained an accuracy of 99.9% for the genitourinary syndrome of menopause dataset and 99.69% for the UCSD dataset. In [59], an application of ViT is presented for fault diagnosis, an average accuracy of 99.9% was achieved considering the 1D-ViT. Besides the accuracy, this model has a low number of floating point operations compared to other CNN structures.

## 3. Materials

The HMDB51 is widely used in the literature [60,61,62]; it is small and has a high noise rate. Small sets can lead to overfitting, making the main objective of the job difficult. It comprises 6849 videos with 51 action classes and at least 101 clips per class. Most of these videos are taken from movies. However, a part comes from YouTube (a public video repository). Furthermore, it is one of the most widely used datasets in the research community for benchmarking state-of-the-art video action recognition models. The classes of the HMDB51 dataset are divided into five groups [12].

(i) General actions related to the face (talking, laughing, and smiling);
(ii) Facial actions with objects (eating, drinking, smoking);
(iii) General body movements (clapping, climbing stairs, jumping, sitting);
(iv) Body movements interacting with objects (kicking, dribbling, pedaling, shooting, and hitting);
(v) Body movements with human interactions (hug, kiss, and greet).

In addition, there are metadata available, along with the videos, with information on the selection of test data, information about the point of view of the cameras, the presence or absence of camera movement, quality, and the number of agents acting [63].

## 4. Methods

This section describes the 3D ResNet and 2D ViT models applied in this paper. These CNNs are used as backbones for the classification task, and the DINO (self-DIstillation with NO labels) is considered to enhance the performance of these structures. DINO is a model developed by Facebook (Meta) applied for self-supervised vision using transformers [64].

DINO focuses on training vision transformers using two main components: clustering and contrastive learning. The first step is to cluster the representations (embeddings) produced by the vision transformer. This involves grouping similar representations and creating clusters that capture different visual patterns in the data. The clustering step helps to provide structure and organization to the learned representations [64].

After clustering, the DINO method employs contrastive learning to refine the representations further. Contrastive learning is a technique where positive and negative pairs of samples are created to encourage the model to bring similar samples closer and push dissimilar samples apart in the embedding space. By doing so, the model learns to discriminate between different visual patterns and improve the overall quality of the representations. The combination of clustering and contrastive learning in this method allows the vision transformer to learn meaningful visual representations in a self-supervised manner [64].

### 4.1. 3D ResNet

ResNet is a popular CNN architecture for image recognition, which utilizes skip connections to avoid the vanishing gradient problem during training. Skip connections allow information from previous layers to be directly passed to deeper layers, improving the flow of gradients, and facilitating deep network training. Three-dimensional ResNet builds upon this architecture by adding an extra dimension to the input data. It is used to process 3D spatial–temporal data such as video frames or medical images, where each image is a 3D volume that changes over time [65].

**Figure 3:**
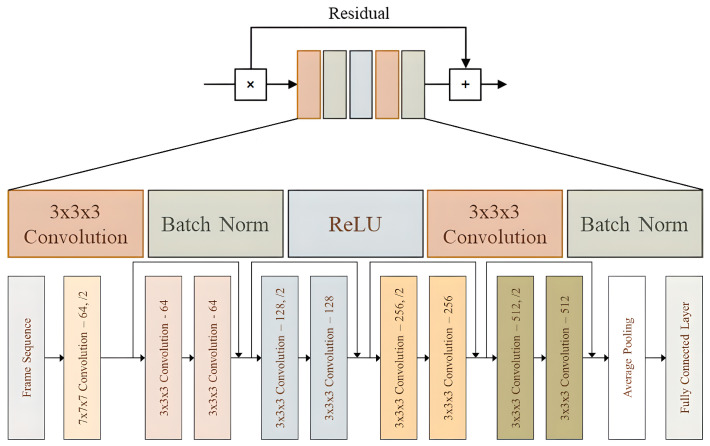
High-level overview of Resnet 3D.

The architecture of 3D ResNet (Figure 3) consists of multiple layers, each of which includes a series of 3D convolutional layers, followed by batch normalization and a nonlinear activation function. The convolutional layers extract features from the 3D input data, and the batch normalization layer normalizes the feature maps to improve the stability and convergence of the training process. The activation function introduces nonlinearity to the output of the convolutional layer [66].

The key innovation of 3D ResNet is using residual blocks, which comprise multiple convolutional layers with skip connections that enable information to bypass some of the layers. This helps mitigate the vanishing gradient problem that can arise in deep neural networks [67]. One of the most popular 3D ResNet architectures is 3D ResNet-50, which has 50 layers and has been widely used in various applications such as action recognition, medical image segmentation, and 3D reconstruction [68].

Thus, 3D ResNet is a powerful neural network architecture for processing 3D spatial-temporal data. By incorporating skip connections and residual blocks, 3D ResNet can effectively handle the challenges of training deep neural networks and has achieved state-of-the-art performance on various 3D data tasks. It was created to process the time dimension along with the image’s width and height [69]. The pre-training phase is performed on large datasets so that fine-tuning is performed on smaller sets. However, the main difficulty of this network is the number of parameters needed to be trained, often being an order of magnitude greater than in bi-dimensional.

In this architecture, the entire hierarchy and relationships between space and time are up to the network to create and discover; it does not need other inputs, such as optical flows and other variables. Furthermore, there are no additional steps in the sequence of the network; the input is processed, and the final output is generated, also called an end-to-end network. However, so that training does not generate overfitting, a large volume of data is needed, a fact that has become possible with new sets such as Kinetics [70].

**Figure 4:**
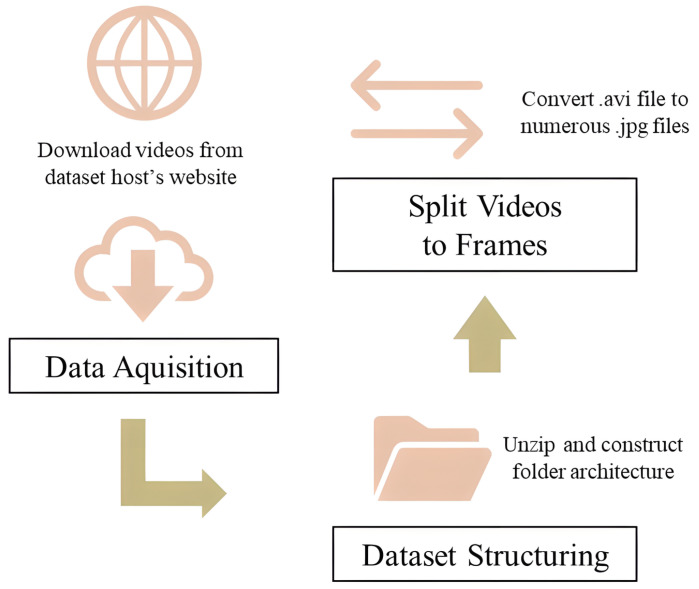
A flowchart of the dataset preparation phase for 3D ResNet.

**Figure 5:**
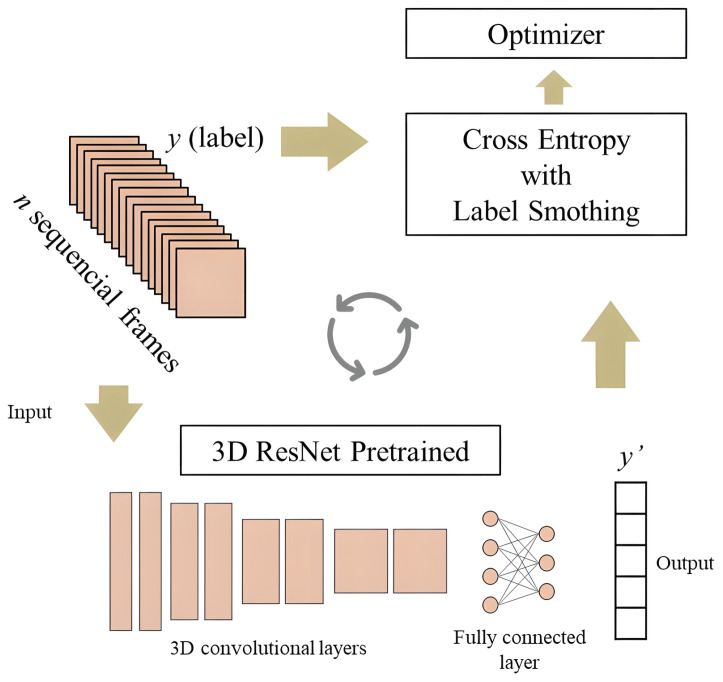
A flowchart of the training process phase for pre-trained 3D ResNet.

Often these architectures become the basis of future models, with pre-trained parameters allowing fine adjustments and small architectural changes to achieve other goals. Figure 4 shows the main step using the 3D ResNet architecture, and Figure 5 presents its training process. Hara et al. [39] trained 3D ResNet models with the Kinetics dataset [70] and the moments in time dataset [71]. This pre-trained model was fine-tuned with HMDB51, and, additionally, the loss function used was cross-entropy with label smoothing.

Label smoothing is a regularization technique that is employed to improve the generalization ability and mitigate overfitting in classification tasks. By modifying the target labels during the training procedure, it instills a sense of ambiguity in the model regarding the definitive labels. This prompts the model to consider the complete probability distribution of the entire class, rather than solely emphasizing the highest probability. As a result, the model demonstrates an enhanced capacity to extrapolate findings to various scenarios and displays increased resilience to disturbances present in the training dataset [72].

### 4.2. Two-Dimensional Vision Transformer

The ViT is a recent approach to computer vision that builds upon the success of the Transformer architecture in natural language processing [73]. Traditional computer vision approaches rely on CNNs to extract features from images, but ViT takes a different approach. Instead of using convolutions, ViT splits the image into a grid of patches, which are then flattened and fed into a Transformer network. ViT’s input is a sequence of patches, rather than a single image, and the Transformer is used to model the relationships between the patches.

ViT consists of two main components: the patch embedding and the Transformer. The patch embedding is responsible for converting each patch into a vector representation that can be fed into the Transformer. This is typically performed using a linear projection layer, which maps each patch to a vector with a fixed dimensionality. The Transformer is then used to model the relationships between the patch embeddings. The Transformer consists of a series of self-attention layers, allowing the network to focus on different parts of the input sequence selectively. The output of the Transformer is a sequence of feature vectors, which can be used for classification or other downstream tasks.

**Figure 6:**
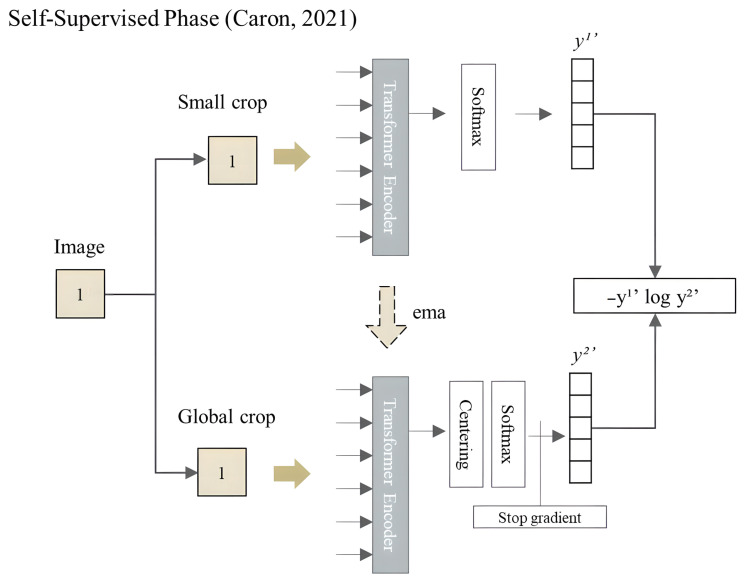
Self-supervised phase of 2D ViT; Caron, 2021 [64].

A key advantage of ViT is its ability to scale to large image sizes, which is difficult for traditional CNN-based approaches. ViT has achieved state-of-the-art performance on a number of benchmark datasets, including COCO [74], CIFAR-100 [75], and ImageNet [64]. Caron et al. [64] applied self-distillation to train a 2D ViT with the ImageNet dataset. An input image was cropped into a small and a global section; each one passes through a different net with the same architecture. A logarithmic loss function was applied between two outputs (y${}^{1^{\prime }}$ and y${}^{2^{\prime }}$), the small section net was trained, and this learning was transferred to the other net by exponential moving average, see details in Figure 6.

**Figure 7:**
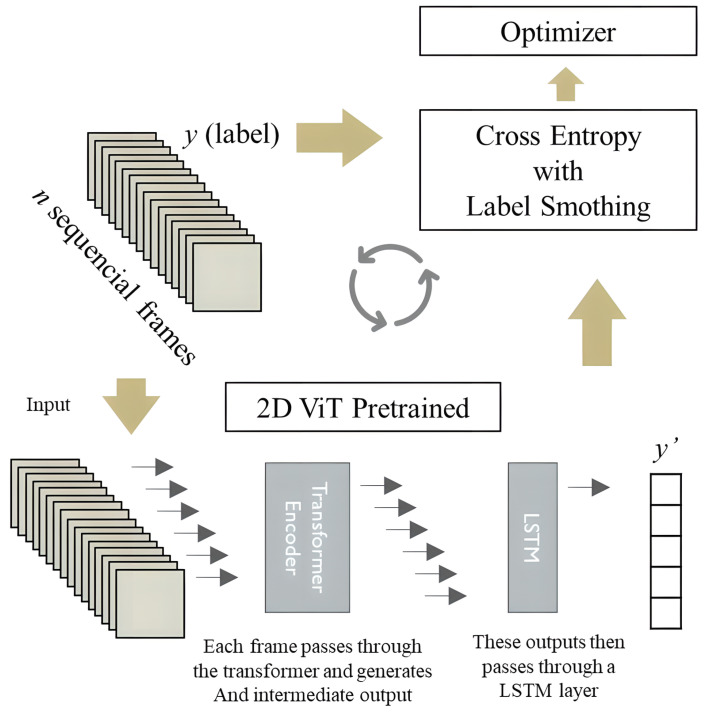
Flowchart for pre-trained 2D ViT. Dataset preparation phase.

To enhance the temporal modeling capabilities of the pre-trained 2D ViT, a fine-tuning approach was employed by replacing the classifier method with an LSTM layer. This LSTM layer effectively captures the memory of all inputs from the video segment previously processed by the 2D ViT, generating a corresponding output. A cross-entropy loss function with label smoothing was applied to optimize the classifier parameters during the training process. For further information and a detailed methodology breakdown, please refer to Figure 7. The procedure of the dataset preparation phase for the pre-trained 2D ViT is equivalent to the procedure for 3D ResNet, which is presented Figure 4.

During the training process, the first step involves loading the annotation file, which contains information about image files and their corresponding labels for each video, thereby constructing the dataset object. Following this, the target model is initialized, and its parameters are either randomly initialized or loaded from a pre-trained model. During each epoch, video segments consisting of “n” sequential frames are transformed, passed through the model, and an output is generated.

**Figure 8:**
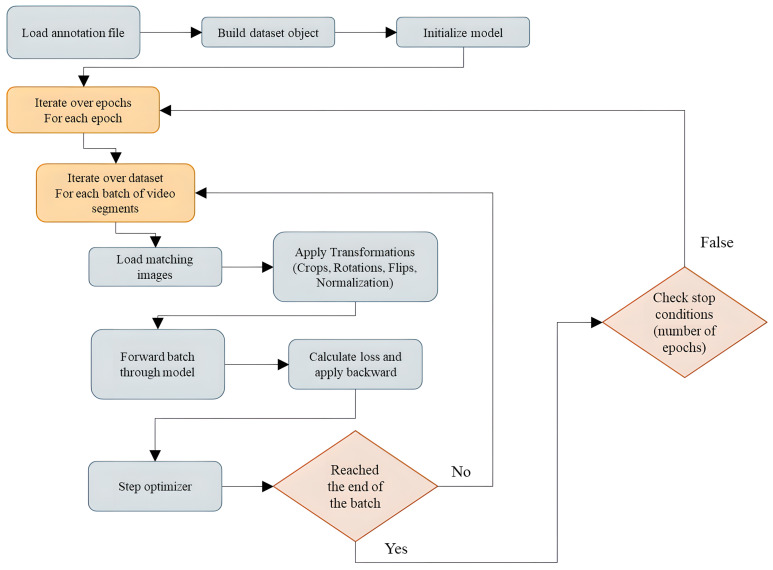
Flowchart of the proposed approach used in this study.

Subsequently, a loss function in the form of cross-entropy with label smoothing is applied, and the model’s parameters are optimized. This iterative process is repeated until all the batches have been processed and the previously planned number of epochs is reached. For a more detailed understanding of the proposed approach, please see Figure 8, which visually represents the approach’s flowchart.

### 4.3. Pre-Processsing and Metrics

We utilized the “normalize” function in the Torchvision transforms package to perform image normalization. This function applies the following normalization procedure:

(1) $$\begin{array}{c} \mathrm{output}=\frac{\mathrm{input}-\mathrm{mean}}{\mathrm{standard}\;\mathrm{deviation}}. \end{array}$$

Cross-entropy loss is a commonly used loss function in machine learning for classification tasks. It measures the difference between the target variable’s predicted and true probability distributions. The cross-entropy loss, or cost function, used to train the model was calculated as follows:

(2) $$\begin{array}{cc} \; & l\left(x,y\right)=L={\left\{l_{1},\cdots ,\;l_{N}\right\}}^{T}, \end{array}$$

(3) $$\begin{array}{cc} \; & l_{n}=-w_{y_{n}}\log\frac{\exp\left(x_{n,y_{n}}\right)}{\sum_{c=1}^{C}\exp\left(x_{n,c}\right)}\cdot 1 \end{array}$$

where *x* is the output of the model, *y* is the target, *w* is the weight, *C* is the number of classes, and *N* is the dimension of the batch.

Accuracy is a widely used metric in classification tasks that measures the proportion of correctly classified instances out of the total number of instances. For accuracy, first, the prediction vector (*p*) is compared to the ground-truth (*Y*). If *p* = *Y*, then 1; otherwise, 0.

(4) $$\begin{array}{c} Acc=\frac{\sum_{i=1}^{n}\left(p_{i}=Y_{i}\right)}{n}. \end{array}$$

## 5. Results

In this section, the results of the performed experiments are presented. The main objectives of these experiments were to test and train deep learning architectures and apply a semi-supervised learning method to the HAR task to overcome the problem of a lack of labeled data. The application of label smoothing, a technique used to reduce the noise in the dataset, was also analyzed. This study used a Dell^®^ Gaming G5-5590-A25B notebook (Dell, Round Rock, TX, USA) with Intel^®^ Core i7 9th generation processor (Intel, Santa Clara, CA, USA), an NVIDIA^®^ GeForce GTX 1660 Ti graphics card (NVIDIA, Santa Clara, CA, USA) with 6 GB dedicated, and 16 GB of random access memory.

After obtaining the data, the frame rate per second (fps), width, height, and duration were scanned. Then, the videos were processed and partitioned in each frame; a 10 s video with 30 fps was partitioned into 300 images, preparing the set to be processed in the algorithm flow. This process was performed for the dataset HMDB51. The video dataset was organized in a structured folder architecture and prepared to run the machine learning models during training. Thus, all videos were split into frames and saved as images.

In each epoch, the batches of video segments with n sequential frames pass through the model and generate an output. Supervised and semi-supervised learning techniques were tested with twenty and eight different configurations applied to HMDB51. However, in the first one, only the best results are presented, either using or not using labeling smoothing.

### 5.1. Supervised Learning

HMDB51 has a certain degree of noise and was used for label smoothing. In this approach, an error factor is inserted in the loss calculation step; considering the batch average at each iteration, a small disturbance is added in network training. This way, problems such as wrong labels and bad and/or noisy data are minimized. For the execution of this experiment, 30 epochs were used. Three-dimensional ResNet was fine-tuned like in Hara et al. [39] with a learning rate of 0.1, a time step equal to four, and a batch equal to eight. Random temporal selection, horizontal inversion, and cut were used.

A multi-step learning rate scheduler (8, 16, 24, and 27) and a classifier with a fully connected layer were used. For this experiment, 31 runs were performed for each view of the HMDB51 datasets (1, 2, and 3), and an average between the views was obtained. The vision of the sets is nothing more than different combinations of videos for training and validation. However, the integrity of the dataset always remained the same for all of them.

Table 2 shows the results of the experiments from 3D ResNet applied to the HMDB51 with or without label smoothing. The performance metric used in this study is accuracy (Acc), and when applying label smoothing there was a drop of approximately eight percentage points, which is reflected in the training loss gain. Comparing the validation loss, there was a reduction of approximately two units.

There was a slight loss in training and a greater reduction in validation accuracy. However, the validation loss function was superior using label smoothing, suggesting greater potential for generalization. As much as label smoothing improves part of the overall results of the network, reflected in the loss of validation, classification is the main objective, so this technique will not bring gains to maximize accuracy. The values presented follow the mean ± standard deviation format of the 31 runs.

### 5.2. Semi-Supervised Learning

Semi-supervised learning executions were performed based on Caron et al. [64]. Thus, two pre-trained networks were applied to recognize human actions, 2D ResNet 50 and 2D ViT. The training process was conducted in an unsupervised manner; that is, the image labels were not used during the training process, only the content itself. It is worth noting that these architectures were developed to work with a single image, so they were adapted for video processing. Each video frame enters the network and generates a set of features that are grouped by video segment and classified into different actions.

The details of the different architectures applied in a database are described in Table 3. Variants 1 to 5 used the 2D ResNet 50 as the base architecture with a batch equal to eight, while variants 6 to 8 used 2D ViT architecture with a batch of 16. Temporal grouping and LSTM were used in the classifier to adapt the 2D network to the 3D scenario. The runs were performed with the following settings: 30 epochs, cut centered on images with dimensions of 224 × 224 pixels, random horizontal inversion, resizing values, standardization, conversion to tensors, and random temporal selection.

Table 4 displays the outcomes of the experiments conducted with the variants outlined in Table 3. In this case, we only present the most promising results obtained from the study, which are the DINO${}_{6}$ to DINO${}_{8}$ variants that outperform the 3D ResNet results presented in Table 2. These results indicate that 2D ViT architectures have high potential in this task.

Table 5 presents a comparison of our proposed hybrid method with two other self-supervised pre-trained models applied to the human activity recognition problem and trained using HMDB51. Our model outperformed the odd-one-out model [76] by 1.1 percentage points and the order prediction network [77] by 4.4 percentage points.

### 5.3. Discussion

Training small datasets could be a hard task as they are difficult to train from scratch, and so they are likely to overfit. HMDB51, with approximately 7k videos and 51 classes, is a small set and, beyond those points, it has noisy labels [78]. Cross-entropy with a label smoothing technique was applied to overpass the last observation. To test the hypothesis that a label smoothing process would achieve a better performance, a 3D ResNet 50 pre-trained by [39] was used. The results found that the model without label smoothing performed better in terms of the training and validation accuracy; however, the model with label smoothing obtained a loss function value 17% lower, indicating a slight trend to generalize better.

In this work, a self-supervised pre-trained network [64] was applied to the HAR task to overcome this barrier. The use of four temporal steps on variant DINO${}_{7}$ brought higher training accuracy; however, the one temporal step on variant DINO${}_{8}$ led to a 0.9 percentage point above the previous one. This indicates that four temporal steps could better model training data while one temporal step achieves superior generalization. Comparing the ViT model using only a fully connected layer, on variant DINO${}_{6}$, with the ViT model using an LSTM layer, on variant DINO${}_{8}$, the LSTM outperformed by 1.7 percentage points, indicating a better aggregation of the temporal information.

## 6. Conclusions

In recent years, data fusion, deep learning approaches, and a combination of models have been widely studied and applied in HAR. Deep learning approaches based on CNN and LSTM have demonstrated remarkable success in HAR. This paper investigated two classifier systems for HAR based on a 3D CNN and a hybrid 2D ViT with LSTM both applied to the HMDB51. The classification results using a 3D ResNet 50 with a fully connected layer and 2D ViT with LSTM demonstrated promising performance in the HMDB51. It obtained 96.7 ± 0.35% and 41.0 ± 0.27% for accuracy scores in the train and test phases, respectively.

In future research, we intend to examine different deep learning architectures such EfficientNet [79] and NASNet (Neural Architecture Search Network) [80] for ensemble learning design combined with feature engineering approaches and the proposed hybrid CNN and LSTM approach in this paper. In addition, we should test the proposed hybrid method on longer and more complex datasets to measure its full capabilities better.

## Figures and Tables

**Table 1:** Dataset used for pre-workout of 3D CNN for group 1.

Pre-Workout Set	No. Articles	%	Authors
Sports 1M	7	41	[38,39,40,41,42,43,44]
Kinetics	3	18	[21,37,42]
Fudan-Columbia Video Dataset	1	6	[45]
Without pre-workout	6	35	[27,46,47,48,49,50]

**Table 2:** Results to the HMDB51 using 3D ResNet.

Label Smoothing	Train Acc (%)	Validation Acc (%)	Train Loss	Validation Loss
No	75.26 ± 0.59	48.9 ± 1.11	2.43 ± 0.10	11.46 ± 0.65
Yes	73.06 ± 0.67	40.84 ± 1.37	3.45 ± 0.06	9.48 ± 0.40

**Table 3:** Architectures applied to HMDB51 using a semi-supervised learning approach.

Variant	Classifier	Learning Rate	Time Step	Method
DINO${}_{1}$	3× Convolutions + Medium Grouping + FCN	0.001	1	temporal grouping
DINO${}_{2}$	3× Convolutions + FCN	0.1	1	temporal grouping
DINO${}_{3}$	1× Convolutions + FCN	0.1	1	temporal grouping
DINO${}_{4}$	2× FCN	0.1	1	temporal grouping
DINO${}_{5}$	1× FCN	0.1	1	temporal grouping
DINO${}_{6}$	1× FCN	0.1	1	temporal grouping
DINO${}_{7}$	LSTM + 1× FCN	0.1	4	2D with LSTM
DINO${}_{8}$	LSTM + 1× FCN	0.1	1	2D with LSTM

**Table 4:** Results to the HMDB51 using 2D ViT.

Variant	Train Acc (%)	Validation Acc (%)	Train Loss	Validation Loss
DINO${}_{6}$	92.3 ± 0.43	40.2 ± 0.30	70.44 ± 0.10	1819.66 ± 0.12
DINO${}_{7}$	96.7 ± 0.35	41.0 ± 0.27	0.15 ± 0.07	2.95 ± 0.23
DINO${}_{8}$	87.1 ± 0.40	41.9 ± 0.29	0.44 ± 0.04	2.55 ± 0.41

**Table 5:** Comparing self-supervised pre-trained models for HAR using HMDB51.

Method	Accuracy (%)
Order Prediction Network pre-trained w/ UCF101 [77]	37.5
Odd-one-out pre-trained w/ ImageNet [76]	40.8
2D ViT + LSTM pre-trained w/ ImageNet (Present study)	41.9

## Data Availability

The dataset used in the experiments of this paper is confidential.
